# Analytical techniques for nucleic acid and protein detection with single-molecule sensitivity

**DOI:** 10.1038/s12276-025-01453-w

**Published:** 2025-05-01

**Authors:** Soochul Shin, Juyoung Kim, Eunho Song, Sun Han, Sungchul Hohng

**Affiliations:** Department of Physics and Astronomy, Institute of Applied Physics, Seoul National University, Seoul, Republic of Korea

**Keywords:** Cancer screening, Cancer screening

## Abstract

Nucleic acids and proteins serve as crucial biomarkers used in the diagnosis, prognosis and therapy monitoring across various diseases. Traditionally, these biomarkers are identified at an ensemble level following the amplification of target or signaling molecules. However, these methods have limitations in addressing contemporary challenges in molecular diagnostics, such as low sensitivity, specificity, throughput and imprecise quantification. To overcome these limitations, various analytical techniques offering single-molecule sensitivity have been developed. Here we aim to provide a concise overview of historically notable and potentially promising analytical techniques to detect nucleic acids and proteins with single-molecule sensitivity. Our focus is on their potential in liquid biopsy, delineating their strengths and weaknesses, providing insights into their ability to revolutionize biomarker analysis and paving the way for more advanced technologies.

## Enhancing liquid biopsy accuracy through single-molecule analysis

Biomarkers are molecules that aid in diagnosing and monitoring diseases. This article explores advanced techniques for detecting proteins and nucleic acids with single-molecule sensitivity. These methods include target amplification approaches, such as digital polymerase chain reaction and next-generation sequencing, signal amplification approaches, such as digital enzyme-linked immunosorbent assay, and bona fide single-molecule analytical techniques, which utilize innovative detection schemes without amplification, such as single-molecule fluorescence imaging. These cutting-edge technologies enable earlier and more precise disease detection compared with the traditional methods. However, challenges such as high costs and technical complexity remain. The authors emphasize the need for future research to focus on making these technologies more accessible and practical for clinical use. Single molecule detection techniques hold great potential for advancing medical diagnostics.

This summary was initially drafted using artificial intelligence, then revised and fact-checked by the author.

## Introduction

Any molecule that plays a critical role in physiology has the potential to serve as a biomarker for human diseases. Nucleic acids and proteins, as major biomolecules in the human body, are among the most crucial biomarkers currently utilized in medicine. Specifically, nucleic acids and proteins are instrumental in diagnosing various cancers, neurodegenerative diseases and infectious diseases. They are also used for prognosis, therapy selection and monitoring.

Historically, the Wassermann test, developed in 1906 for the detection of syphilis antibodies, marked an early use of protein biomarkers in diagnostics for infectious diseases^1^. The use of proteins in oncology also has a rich and long history dating back to the mid-nineteenth century, marked by the discovery of Bence Jones protein in the urine of a patient with multiple myeloma^2^. However, proteins began to be extensively utilized as tumor markers following the discovery of alpha-fetoprotein associated with hepatocellular carcinoma^3^ and carcinoembryonic antigen elevated in the blood of individuals with various types of cancer in the 1960s^4^, culminating in the discovery of prostate-specific antigen in the year 1979^5^. These proteins are currently employed for screening, diagnosis, monitoring treatment responses, detecting recurrence and assessing prognosis in related cancers. The protein biomarkers with clinical potential in other diseases include amyloid-beta (Aβ), tau proteins and neurofilament light chain for neurodegenerative diseases^6^ and troponin for cardiovascular diseases^7^.

The use of protein biomarkers in medicine advanced alongside the development of modern analytical tools for protein detection, such as immunoassays. For instance, enzyme-linked immunosorbent assay (ELISA) and related techniques, developed in the 1970s, have enabled the sensitive and specific detection of protein biomarkers^8^. In ELISA, target proteins are immobilized on a solid surface. These target proteins are then recognized by an antibody linked to an enzyme, which generates a chromogenic signal by continuously converting its substrates in the detection chamber. This signal amplification approach provides high sensitivity, making ELISA a widely used method in clinical diagnostics.

Nucleic acid biomarkers encompass a variety of molecules, including DNA, messenger RNA and noncoding RNAs. These molecules play diverse roles as genetic materials and messengers and regulators in gene expression, making them an important group of biomarkers. Their clinical application became feasible with the development of the polymerase chain reaction (PCR) in the 1980s^9^. PCR generates millions of copies of a target molecule through repeated enzymatic amplification, achieving high specificity and sensitivity. As a result, PCR has become the gold standard for nucleic acid detection.

Conventional analytical techniques for protein and nucleic acid detection, such as ELISA and PCR, have historically played a crucial role in establishing molecular diagnostics and remain standard techniques today. As molecular diagnostics aims to tackle more complex challenges, however, there is a growing demand for more advanced diagnostic techniques with improved accuracy and throughput. For example, liquid biopsy aims to detect cancer by analyzing biomarkers present in biofluids such as blood, urine or saliva^10^. Given that biomarkers in these biofluids are typically present in minute amounts and alongside similar molecules, enhanced analytic sensitivity and specificity are essential. Achieving high diagnostic accuracy requires quantifying multiple biomarkers efficiently and cost-effectively within a reasonable timeframe.

To address these challenges, various techniques with single-molecule sensitivity have been developed. In the realm of molecular diagnostics, single-molecule measurements provide several advantages over ensemble techniques such as conventional ELISA and PCR^11^. They can detect rare biomarkers and mutations present in low concentrations that might be overlooked by ensemble averaging, thereby enhancing sensitivity. Single-molecule methods also offer real-time analysis capabilities, enabling the observation of dynamic processes and interactions as they unfold. This kinetic information can distinguish between closely related biomarkers or variants with greater specificity compared with ensemble approaches. Moreover, single-molecule techniques ensure precise quantification of biomarkers, delivering accurate measurements of biomarker concentrations without the variability inherent in ensemble averages. They also enable the study of molecular heterogeneity within a single sample, facilitating efficient multiplexed detection. Overall, single-molecule measurements offer superior sensitivity, specificity, precision and throughput, thereby proving invaluable in advancing molecular diagnostics, biomarker discovery and personalized medicine.

**Fig. 1:**
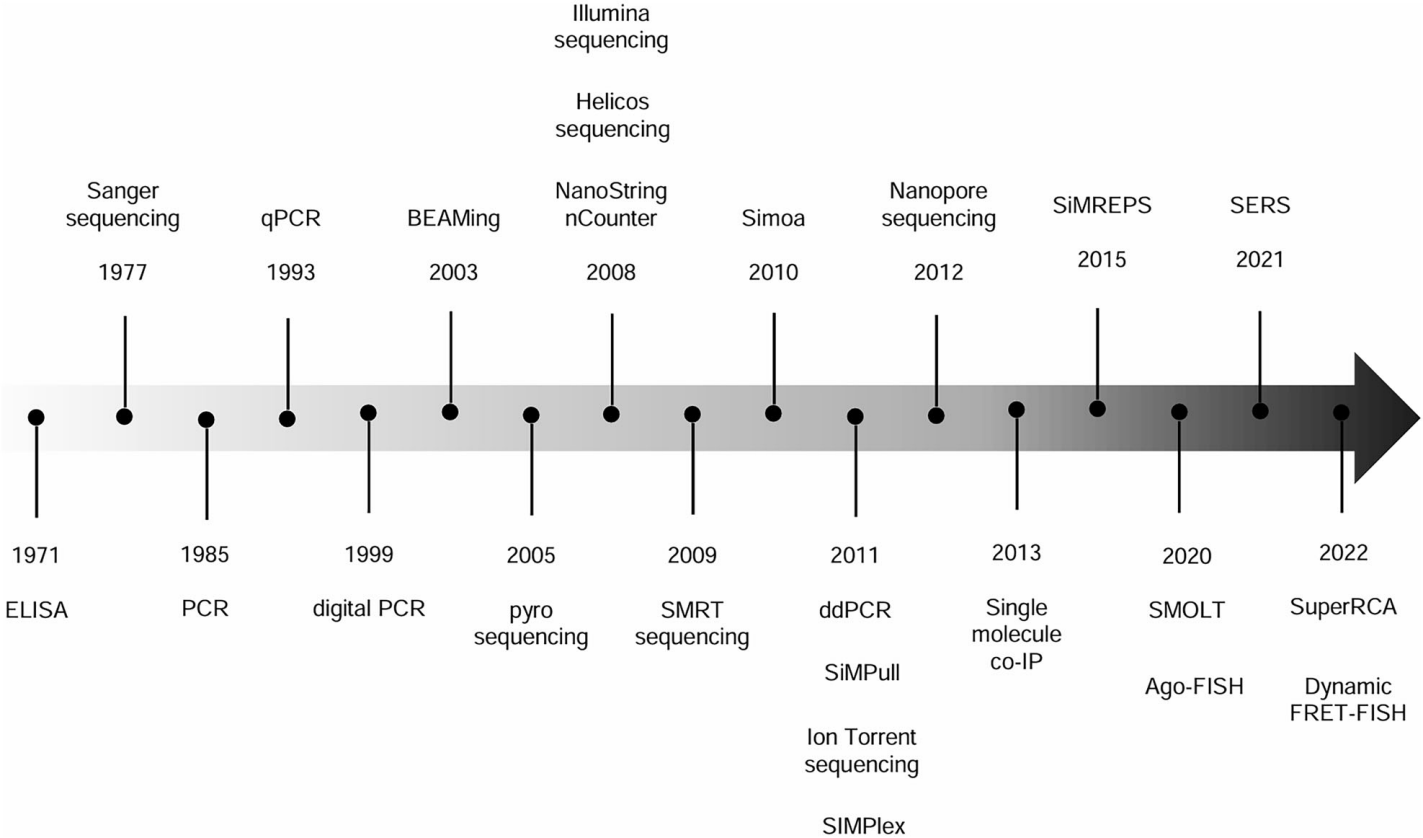
Key milestones in the advancement of molecular diagnostic tests. A timeline illustrating the progression in the development of molecular diagnostic tests reviewed in this study, highlighting notable milestones that have led to the achievement of single-molecule sensitivity.

This article explores various strategies developed to achieve single-molecule detection sensitivity in nucleic acid and protein detection (Fig. 1 and Table 1), focusing on their potential in liquid biopsy and delineating their strengths and weaknesses. Importantly, this Review does not aim to provide a comprehensive overview of all reported methods capable of single-molecule detection but rather presents selected examples that illustrate conceptual advancements in this field.

**Table 1:** A summary of molecular diagnostic techniques with single-molecule detection sensitivity.

Technique	Target	Method	Detection point	Multiplexing	Sensitivity^†^
Digital PCR	Nucleic acid	Target amplification	End point	Low	0.1%
BEAMing	Nucleic acid	Target amplification	End point	Low	0.01%
SuperRCA	Nucleic acids	Target and signal amplification	End point	Low	0.001%
Pyrosequencing	Nucleic acid	Target amplification	End point	High	1%
Ion Torrent sequencing	Nucleic acid	Target amplification	End point	High	1%
Illumina sequencing	Nucleic acids	Target amplification	End point	High	0.1%
nCounter	Nucleic acid	Bona fide	End point	High	1 fM
SiMREPS	Nucleic acid	Bona fide	Real time	Medium	0.0001%
Ago-FISH	Nucleic acid	Bona fide	Real time	Medium	0.008%
Dynamic FRET-FISH	Nucleic acid	Bona fide	Real time	Medium	
SiMPull	Protein	Bona fide	Real time	Medium	1 pM
SIMPlex	Protein	Bona fide	Real time	Medium	1 pM
Single-molecule co-IP	Protein	Bona fide	Real time	Medium	1 pM
Simoa	Protein	Signal amplification	End point	Low	<1 fM
SERS	Protein	Bona fide	End point	Medium	
SMOLT	Nucleic acid	Bona fide	End point	Medium	<1 fM
Helicos sequencing	Nucleic acid	Bona fide	End point	High	
SMRT sequencing	Nucleic acid	Bona fide	Real time	High	
Nanopore sequencing	Nucleic acid	Bona fide	Real time	High	

^**†**^When possible, the lowest detectable VAFs of point-mutant ctDNAs are provided. In other cases, the lowest detectable concentrations are specified.

## Compartmentalized amplification of single nucleic acid targets

To achieve single-molecule sensitivity in biomarker detection, one effective approach involves partitioning the sample at the level of single molecules and performing target amplification in the separate partitions. Given that compartment size limitations exist, it is crucial to maintain a low biomarker concentration to ensure some compartments are devoid of biomarkers while others may contain one or more. The efficiency of target amplification is vital in generating a detectable signal postamplification of a single biomarker. Without a current method to amplify protein targets, this approach is only appropriate for nucleic acid biomarkers. In this study, we explore various approaches to its implementation.

### Digital PCR

**Fig. 2:**
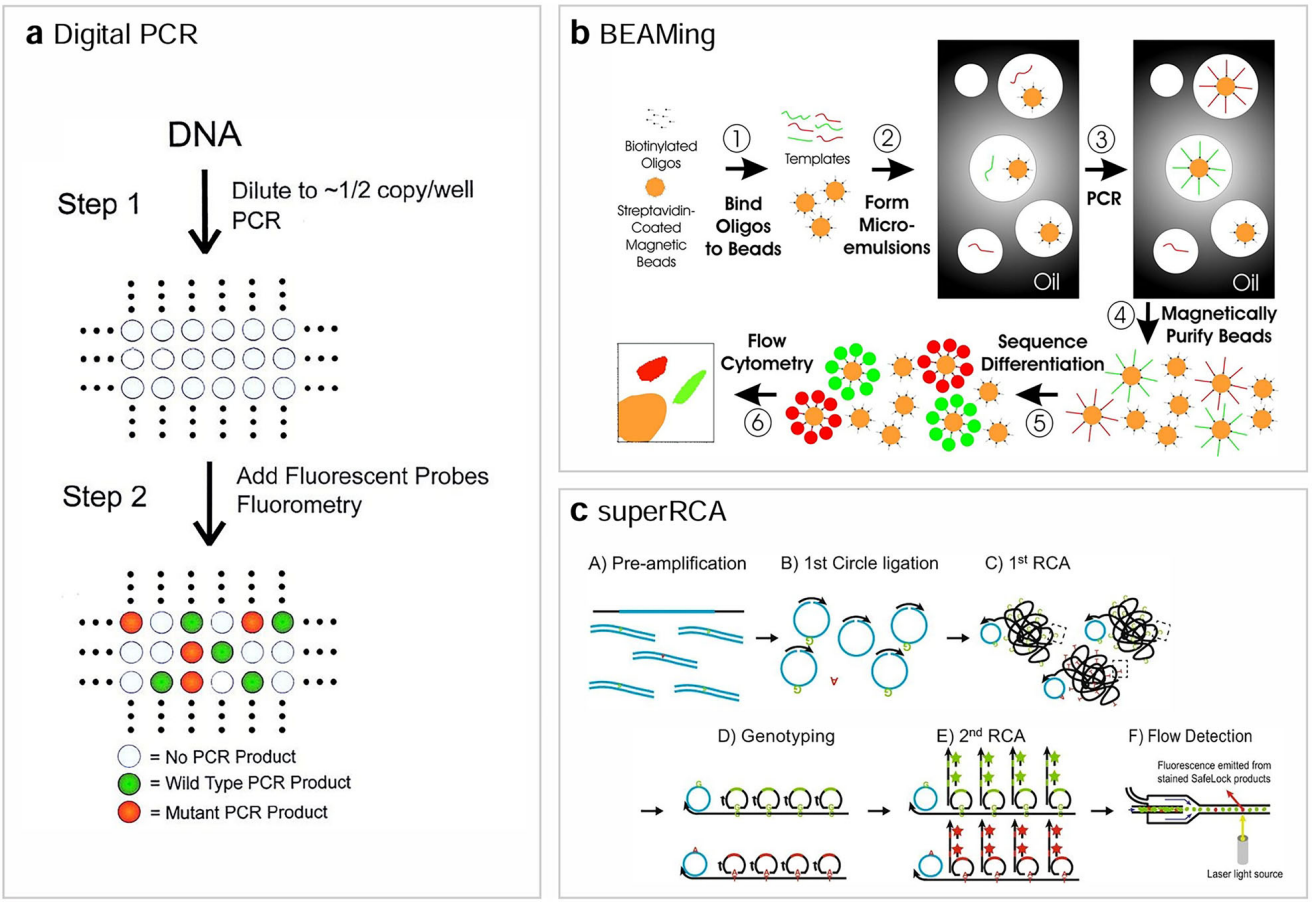
Compartmentalized amplification of single nucleic acid targets. **a**–**c** The schematics of digital PCR^12^ (**a**) BEAMing^17^ (**b**) and SuperRCA^20^ (**c**).

The term ‘digital PCR’ was officially coined in the year 1999^12^, but the technique has a much longer prehistory. Shortly after the introduction of PCR in 1985, the potential for single-molecule detection using PCR was recognized and harnessed, leading to the development of a technique known at the time as single-molecule PCR or limit dilution PCR^13^. In this analysis method (Fig. 2), the mixture comprising target nucleic acid, primers and a probe is segmented into numerous equally sized partitions. After PCR amplification, partitions with target DNA can be differentiated from those without target DNA based on their fluorescence signal.

The quantification of the target nucleic acid copy number per partition is determined by the number of positive partitions and the total partition count using a Poisson model. This ability to assess target concentration without the need for a preestablished calibration curve is a key advantage of digital PCR over traditional quantitative PCR (qPCR). In qPCR, the concentration of a target nucleic acid is determined by referencing a calibration curve, which is generated from experiments involving a dilution series of the target nucleic acid. However, the efficiency of qPCR amplification can be affected by various factors such as variations in target sequences, primer selection and inhibitory substances in the sample matrix. This variability can lead to inconsistent results, making qPCR a less reliable tool for molecular diagnostics. Furthermore, qPCR may have limitations in accurately quantifying low-abundance targets, particularly in samples with a high background of non-target DNA or RNA. By contrast, digital PCR discriminates partitions with and without a target molecule in a binary manner (on or off), making it much more immune to factors affecting amplification efficiency. In addition, digital PCR is an optimal technique for low-concentration samples. As a result, digital PCR excels in detecting and quantifying rare variants among a large excess of normal sequences, such as in the detection of circulating tumor DNA (ctDNA) in cell-free DNA. In ctDNA detection, a 0.1% variant allele frequency (VAF) was demonstrated using digital PCR compared with a 1% VAF with qPCR.

Despite the obvious advantages of digital PCR over qPCR in molecular diagnostics, its practical utilization in medicine could be realized with advances in microfluidic technology to automatically create tens of thousands or more partitions. Currently, there are primarily two methods used to create partitions: microfluidic droplet generation and microwell arrays^14^. In the first method, called droplet digital PCR, the reaction mixture is emulsified into tiny water-in-oil droplets. Each droplet serves as an individual partition where the PCR reaction can occur independently. This approach generates the largest number of partitions but is time consuming and requires multiple instruments (droplet generator, thermocycler and droplet reader). The variability in droplet size can adversely affect the robustness of the analysis outcome. The second approach involves distributing the reaction mixture into a plate that contains tens of thousands of small wells, with each well acting as an individual reaction chamber. Compared with droplet digital PCR, this approach is more convenient and provides faster analysis results.

Several research papers have highlighted the precise quantification and improved sensitivity offered by digital PCR^15,16^. Nevertheless, despite its advantages, digital PCR faces certain limitations that must be resolved to enable its broader adoption in molecular diagnostics. While digital PCR is generally more robust than qPCR, it can still be affected by inhibitors present in the sample matrix, which may compromise amplification efficiency and result accuracy. Moreover, digital PCR instruments and consumables are typically more expensive than those for qPCR. Similar to qPCR, digital PCR faces challenges in multiplexing, which limits its utility in fields such as oncology where detecting multiple targets from a limited specimen is crucial for accurate diagnosis. Furthermore, digital PCR, similar to qPCR, is sensitive to contamination, necessitating that the diagnostic process be performed in a controlled and protected facility.

### BEAMing

Digital PCR has been demonstrated to surpass qPCR in quantifying rare mutations amidst wild-type sequences, offering an order of magnitude improvement in sensitivity for detecting these mutations (0.1% versus 1% limit of detection (LoD)). However, in patients of early-stage cancer with small tumors, the fraction of ctDNA can be substantially lower than the LoD of digital PCR. To quantify rarer mutations using digital PCR, an increased number of partitions need to be efficiently analyzed. Bead, emulsion, amplification and magnetics (BEAMing) is an advanced digital PCR technique developed to address this challenge^17,18^.

The technology involves converting single DNA molecules into single magnetic beads (Fig. 2b). To achieve this, hundreds of millions of water-in-oil droplets are generated, each potentially containing a single target DNA molecule and a single magnetic bead. Within each droplet, the target DNA is amplified using PCR, resulting in thousands of copies of the original DNA attached to each bead. The beads, now covered in amplified DNA, are extracted from the emulsion using magnetic separation techniques. The number of mutant and wild-type DNA molecules in the population can then be assessed by differentially staining the beads with distinct fluorophores and counting them using flow cytometry. Using BEAMing, a LoD of 0.01% can be achieved, an order of magnitude improvement compared with conventional digital PCR.

The first clinical data were obtained through the application of BEAMing to analyze plasma samples from cancer patients^19^. However, despite its high sensitivity and specificity for detecting rare mutations, it is not widely utilized in molecular diagnostics due to several limiting factors. It is a technically complex and labor-intensive process requiring specialized instruments and highly trained personnel. Consequently, it is costly and time consuming. Moreover, similar to conventional digital PCR, scaling up BEAMing for high-throughput applications is challenging.

### SuperRCA

The super rolling circle amplification (superRCA) is another approach to detect low-frequency mutations in a large background of wild-type DNA^20^. In this technique, DNA sequences of interest, including both mutant and wild-type DNAs, are first enriched using preamplification (Fig. 2c). The amplification products are then converted into DNA circles using ligation. These DNA circles undergo isothermal rolling circle amplification (RCA), producing long single-stranded DNA molecules that contain several hundred to a thousand copies of the complement of each starting DNA circle. The circular padlock probes specific for mutant or wild-type sequences are then annealed to the repeated sequences of the first RCA products, followed by a second round of RCA using the padlock probes. This process, known as SuperRCA, results in a single DNA molecule being converted into a large DNA cluster containing millions of copies of a tag sequence in total. The final products of SuperRCA for mutant and wild-type DNAs are visualized using fluorophore-labeled hybridization probes and counted as individual, brightly fluorescent mutant- or wild-type-specific objects using flow cytometry. Using SuperRCA, 0.001% of LoD for point mutantation detection can be achieved, an order of magnitude improvement compared to BEAMing.

The ultrasensitive mutation detection capability of SuperRCA was effectively employed to monitor the progression of patients undergoing treatment for acute myeloid leukemia by monitoring extremely rare tumor-specific mutations in plasma, including deletions, insertions and single nucleotide variations^20^. Despite the remarkable achievements of the SuperRCA technique, several hurdles must be addressed for it to advance from a promising research tool to a routinely used method in molecular diagnostics, potentially revolutionizing the detection and monitoring of low-frequency mutations in various disease contexts. Notably, SuperRCA involves multiple steps, including preamplification, DNA circle generation, first isothermal RCA, padlock probe annealing, second isothermal RCA, hybridization of fluorescent probes and detection of the final SuperRCA products using flow cytometry. This workflow can be labor-intensive and time consuming, limiting its practicality for high-throughput clinical applications where rapid turnaround times are crucial. It remains to be seen whether the entire SuperRCA process can be automated to streamline the workflow and reduce hands-on time.

### NGS

While PCR and related nucleic acid detection techniques are valuable and widely used in molecular diagnostics for their speed, simplicity and specificity, they are low-throughput technologies that can detect only a small number of predefined genetic regions, primarily reporting the presence or absence of specific genetic variants. However, there are clinical settings where a wide range of genetic variants or comprehensive genetic profiles need to be characterized. For instance, disorders such as cystic fibrosis or muscular dystrophy involve numerous possible mutations in the responsible genes^21^. Cancer also involves complex genetic alterations, including mutations, copy number changes and structural rearrangements, necessitating a comprehensive, high-throughput technique that can detect numerous genetic variants simultaneously.

Next-generation sequencing (NGS), also known as massive parallel sequencing or second-generation sequencing, is a technology that can address this challenge. Compared with Sanger sequencing—also known as capillary sequencing or first-generation sequencing—which processes one DNA fragment at a time^22^, NGS can sequence millions of DNA fragments simultaneously. This remarkable fit can be accomplished through the following steps: (1) generation of sequencing libraries, (2) spatially separated clonal amplification of sequencing libraries and (3) sequencing by synthesis of these amplified DNA templates in a massively parallel fashion. NGS platforms come in various forms, each with unique strategies and technologies to realize the final goal of massively parallel sequencing.

**Fig. 3:**
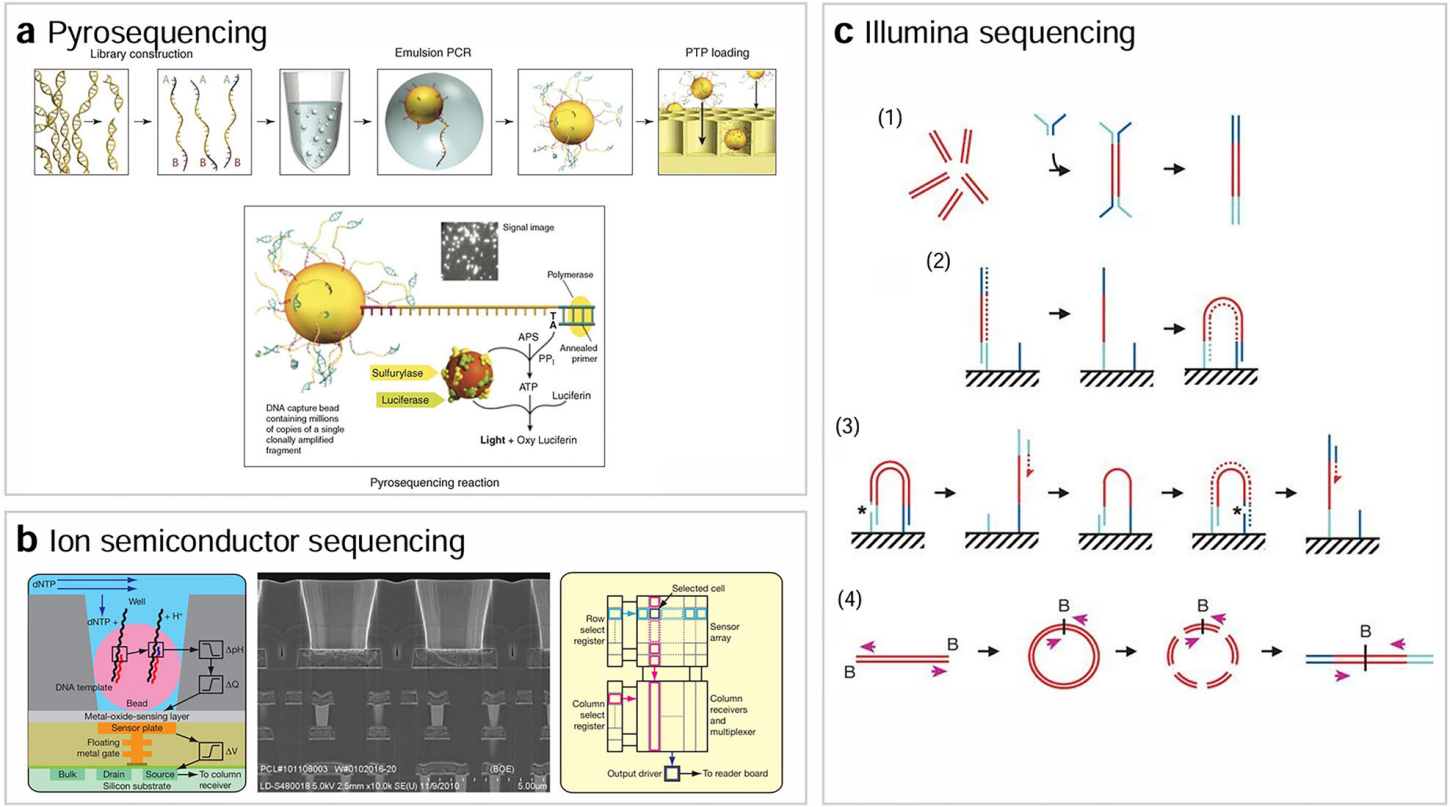
NGS technologies. **a**–**c** The schematics of pyrosequencing^63^ (**a**) ion semiconductor sequencing^24^ (**b**) and Illumina sequencing^25^ (**c**).

The pyrosequencing technology, exemplified by Roche 454 sequencing, involves a series of steps to prepare sequencing libraries^23^ (Fig. 3a). First, extracted DNA is mechanically fragmented into appropriately sized pieces for pyrosequencing. These fragments undergo repair to create blunt ends. Subsequently, specific sequencing adapters are ligated to the blunt-ended DNA fragments. These adapters contain sequences for binding to the sequencing primer and for attachment to beads. Emulsion PCR is then employed for clonal amplification of the sequencing libraries. This process involves placing individual DNA fragments bound to beads into a water-in-oil emulsion with PCR reagents. Through emulsion PCR, millions of copies of the DNA fragment are generated within each emulsion droplet containing a single DNA fragment attached to a bead. For sequencing, the beads carrying amplified DNA fragments are loaded onto a sequencing plate, ensuring they are spatially separated for individual sequencing. A sequencing primer is annealed to the adapter sequence on each DNA fragment bound to the beads, providing the starting point for the sequencing-by-synthesis reaction. The sequencing reaction proceeds cyclically, with each cycle involving the addition of a single type of nucleotide (dATP, dTTP, dCTP or dGTP) to the reaction mixture. The release of pyrophosphate during nucleotide incorporation triggers a series of enzymatic reactions culminating in the generation of chemiluminescence. This light is detected by a camera, producing a pyrogram—a graphical representation of light intensity over time—which correlates with the sequence of the DNA.

Ion semiconductor sequencing, exemplified by Ion Torrent sequencing, shares similarities with pyrosequencing in terms of library preparation and clonal amplification methods^24^ (Fig. 3b). However, they differ in their sequencing detection strategies: ion semiconductor sequencing electronically detects changes in pH caused by the release of H^+^ ions during nucleotide incorporation, whereas pyrosequencing utilizes a chemiluminescence signal generated by luciferase.

The NGS technique developed by Illumina utilizes a sequencing library preparation strategy similar to those of pyrosequencing and semiconductor sequencing, with the addition of an adenine (A) nucleotide to the 3′ ends of repaired DNA fragments^25^ (Fig. 3c). This A-tailing step primes the DNA fragments for subsequent adapter ligation. Adapters contain sequences necessary for amplification and sequencing, serving as priming sites and providing unique indices (barcodes) for multiplexing—where multiple samples are pooled and sequenced together in one run. Next, adapter-ligated DNA fragments are immobilized on a solid surface, and DNA clusters are generated via bridge amplification, amplifying each DNA fragment into thousands of copies per cluster. Sequencing commences with the addition of a single fluorescently labeled nucleotide (dNTP) to the clusters. The nucleotides are added sequentially, with each incorporation event detected by a camera capturing fluorescence emitted from the labeled nucleotide. After detection, a cleavage step removes the fluorescent label, enabling the next cycle of nucleotide addition. The images of the emitted fluorescent signals from each cluster are captured and analyzed to determine the sequence of nucleotides incorporated at each cycle.

The primary advantage of NGS is its high-throughput capability, allowing for the potential detection of all genomic variants, including novel ones. However, the vast amount of data generated by NGS often lacks known clinical relevance. To reduce diagnostic time and cost while improving accuracy, targeted sequencing is commonly performed in clinical applications, with this form of NGS being the most popular technology for liquid biopsy^10^. Despite this strategy, however, the turnaround time from sample collection to result delivery can be much longer for NGS compared with some traditional methods, largely delaying clinical decision-making. While the cost of NGS has decreased, it remains relatively high, affecting its accessibility and widespread adoption. In addition, the detection sensitivity of NGS, with a LoD of 1%, is not sufficient for ctDNA analysis. Utilizing advanced methods such as unique molecular identifier technology can reduce the LoD to 0.1%, albeit with a substantial increase in cost. Even with these advancements, mutation detection of commercial NGS systems remains unreliable when VAF is below 0.5%^26^. There are ongoing efforts to further improve the ctDNA detection sensitivity of NGS by accepting a reduction in its multiplexing capacity. For example, the Safer-Sequencing System (Safer-SeqS) can detect variant frequencies below 1 in 100,000 DNA molecules^27^. This is achieved by introducing identical molecular barcodes into the Watson and Crick strands of template molecules, enriching target sequences with strand-specific PCR and redundantly sequencing the amplification products.

## Compartmentalized amplification of signaling molecules by individual protein targets

Without a target amplification method, one potential strategy for detecting single protein biomarkers involves amplifying signaling molecules produced by individual proteins within isolated compartments. Currently, only one commercial platform employs this approach, known as digital ELISA or single-molecule array (Simoa)^28^. In this technique, antibody-conjugated magnetic beads capture a protein biomarker complexed with a biotin-labeled detection antibody and streptavidin-β-galactosidase. The concentrations of magnetic beads and protein biomarkers are carefully adjusted so that each magnetic bead captures either zero or one protein biomarker. These magnetic bead-protein biomarker complexes are then loaded into spatially separated wells, where the ELISA reaction is conducted individually in each well. Single protein biomarkers can be quantified by detecting localized fluorescence signals from active wells. One advantage of digital ELISA is its ability to lower the LoD to subfemtomolar levels and even subattomolar levels in some circumstances^29^, whereas conventional ELISA typically operates in the picomolar range. Digital ELISA has been utilized in liquid biopsy, such as for quantifying extracellular vesicle levels in patients with early-stage breast cancer^30^. However, a notable limitation of digital ELISA is its extended turnaround time, which must be addressed for broader acceptance in molecular diagnostics.

## Bona fide single biomarker detection

With advances in detector technologies and detection strategies, it is now possible to detect single molecules without amplification. These bona fide single-molecule detection techniques offer advantages in molecular diagnostics over those that rely on the amplification of target biomarkers or signaling molecules. Without the need for amplification, they provide faster diagnoses. Not only is end-point analysis possible but also is real-time monitoring feasible, offering additional information to aid in the discrimination of minute differences. Although they are not yet widely utilized in clinics, these techniques have potential to revolutionize molecular diagnostics. Various approaches are employed, and some representative techniques are reviewed below.

### Fluorescence-based single biomarker detection

Fluorescence is arguably the most convenient and popular method for observing single biomarkers. Since the first observation of single fluorophores in a condensed phase in the year 1990^31^, various approaches for fluorescence imaging at the single-molecule level have been developed. However, high throughput and high speed are critical for molecular diagnostics. To achieve this, target biomarkers are typically immobilized on the surface of the detection chamber and fluorescently labeled either transiently or permanently. Single-molecule fluorescence signals are then detected using total internal reflection fluorescence microscopy equipped with a highly sensitive camera. The selected examples of fluorescence-based single biomarker detection techniques with potential in molecular diagnostics are reviewed below.

**Fig. 4:**
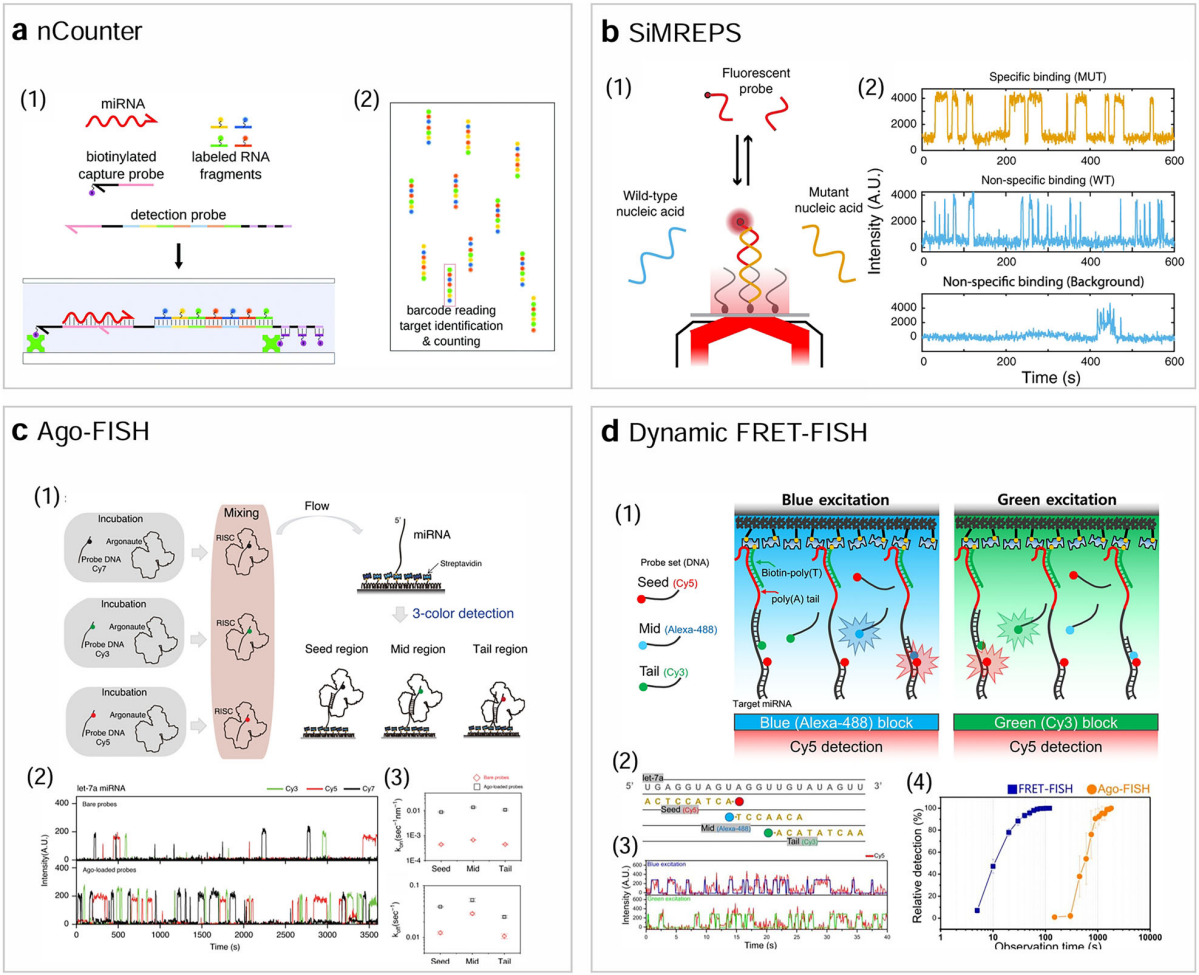
Single-molecule fluorescence techniques for nucleic acid detection. **a**–**d** The schematics of nCounter^64^ (**a**) SiMREPS^65^ (**b**) Ago-FISH^39^ (**c**) and dynamic FRET-FISH^41^ (**d**).

The NanoString nCounter was originally demonstrated to quantify mRNA levels for hundreds of genes simultaneously by counting individual mRNA molecules^32^ (Fig. 4a). In mRNA detection, the target mRNA is hybridized with two probes: a capture probe and a reporter probe. The capture probe is biotinylated for surface immobilization. The reporter probe, in addition to having a region complementary to the target mRNA, contains a single-stranded DNA region annealed to a series of RNA segments, each labeled with a specific fluorophore. The linear order of these differently colored RNA segments, determined via single-molecule imaging, provides a unique barcode for each mRNA. By modifying the design of the capture and reporter probes to interact with different target molecules, the nCounter can be converted into a versatile molecular profiling tool capable of analyzing various nucleic acids, proteins and post-translational modifications.

The nCounter system provides absolute counts of target molecules without the need for amplification, reducing biases and errors associated with amplification and allowing precise quantification. It can simultaneously analyze hundreds of different target molecules in a single reaction, providing comprehensive profiling. The system supports a wide range of applications, including gene expression, miRNA analysis, copy number variation and protein profiling. With these advantages, nCounter is used in a wide range of research. It also holds potential for clinical applications, such as companion diagnostics^33^. However, it also has some notable technical disadvantages. While its multiplexing capability is high, it is lower compared with NGS for some applications. The turnaround time and diagnostic costs of nCounter need to be reduced for widespread application in clinics. The LoD of nCounter in the femtomolar range is not adequate for certain applications. In addition, it does not provide sufficient specificity to detect minute variations such as point mutations.

Single-molecule recognition through equilibrium Poisson sampling (SiMREPS) was originally developed to detect miRNAs with high specificity^34^ (Fig. 4b). miRNAs are short (19–24 nt) noncoding RNAs that post-transcriptionally regulate 30% of protein-coding genes. Due to their small size and high sequence homology, conventional nucleic acid detection techniques such as qPCR and NGS often lack the specificity and accuracy needed for precise quantitation. SiMREPS addresses these challenges by observing transient binding and dissociation kinetics. In SiMREPS, target miRNAs are captured on a surface by base-pairing with partially complementary locked nucleic acids, and transient binding events of fluorescently labeled DNA probes to target miRNAs are monitored using single-molecule imaging. This method provides a distinctive kinetic signature or ‘kinetic fingerprint’, of target-probe interactions, permitting a high discrimination between single nucleotide differences and overcoming the thermodynamic limits of specificity. SiMREPS does not have biases and errors associated with amplification and can more precisely quantify target miRNAs than qPCR and NGS.

The concept of SiMREPS, which achieves high specificity based on kinetic fingerprints, can be generalized to detect various types of biomarker beyond miRNAs including small molecules and ctDNAs^35,36^. In Fab-based SiMREPS, for example, target proteins are immobilized on a surface using a capture antibody (typically an IgG) with a low dissociation constant (*K*_d_)^37^. Fluorescently labeled Fabs with a higher dissociation constant (*K*_d_ ≥ 10 nM) are then introduced into the detection chamber to monitor repetitive binding between the Fab and the immobilized target protein. This method considerably improves the specificity of target protein recognition by eliminating nonspecific signals based on kinetic analysis.

With improved specificity, SiMREPS can detect point mutations in ctDNA with LoD as low as 0.0001% (ref.^38^). While SiMREPS offers high specificity for biomarker detection, several technical challenges limit its widespread adoption. Single-molecule imaging systems are expensive and complex to operate, requiring a high level of technical expertise. The cost of specialized equipment, reagents and skilled personnel makes SiMREPS an expensive technique to implement and maintain. Data acquisition and detailed analysis of single-molecule binding/dissociation events are time consuming, limiting the number of samples that can be processed in a given timeframe. Reproducibility can be an issue due to variability in surface preparation, probe labeling and imaging conditions. Detecting low-abundance targets without amplification is challenging. While incubating large volumes of sample for several hours can achieve a femtomolar range LoD, this approach may not be practical for clinical applications. Scaling up the technique for large sample sizes or routine clinical use is required for broader application and acceptance of SiMREPS in molecular diagnostics.

In the context of biomarker detection using binding/dissociation kinetics, as in SiMREPS, detection time can be reduced by accelerating the binding and dissociation rates of fluorescent probes. The binding of a bare DNA probe to its complementary target is typically a slow process, with a binding time of around 1000 s nM^−1^. To address the lengthy data acquisition time associated with SiMREPS, the Argonaute-based fluorescence in situ hybridization (Ago-FISH) technique was developed^39^ (Fig. 4c). This technique leverages the discovery that a DNA probe preloaded onto Argonaute proteins can locate its target more than 20 times faster and dissociates from the target several times quicker than a bare probe. In Ago-FISH, miRNAs are polyadenylated and then captured on the surface of a detection chip by poly(T) DNAs instead of partially complementary locked nucleic acids. This approach ensures that the entire region of the target miRNA is exposed for detection using multicolor imaging, resulting in high specificity for the entire miRNA sequence. Moreover, multiple targets can be universally captured on the surface using the same DNA, facilitating the detection of multiple targets through probe exchange. Ago-FISH has been demonstrated to detect miRNAs with much higher specificity and speed than qPCR. In addition, Ago-FISH can detect multiple ctDNAs in a short time with greater sensitivity than both qPCR and NGS^40^.

Dynamic fluorescence resonance energy transfer-based fluorescence in situ hybridization (FRET-FISH) employs a different approach to address the slow detection speed of SiMREPS^41^ (Fig. 4d). The binding rate of DNA probes increases linearly with probe concentration; however, in single-molecule fluorescence experiments, increasing the probe concentration indefinitely is not feasible because the background noise from free-floating fluorescent probes overwhelms the single-molecule fluorescence signals from target-binding probes. By detecting only acceptor signals due to FRET, while donor fluorophores are alternately excited, background fluorescence is greatly reduced. This permits the use of much higher probe concentrations in single-molecule imaging. Compared with Ago-FISH, dynamic FRET-FISH can reduce miRNA detection time by 60-fold. Moreover, dynamic FRET-FISH has the advantage of inherently eliminating signals from nonspecific probe binding. While both Ago-FISH and dynamic FRET-FISH address the slow analytical speed of SiMREPS, other technical challenges of SiMREPS, such as high cost and reproducibility issues, remain to be addressed for their widespread adoption in molecular diagnostics.

Single-molecule studies on proteins are typically conducted using purified recombinant proteins from bacterial or other expression systems. However, for diagnostic purposes, it is desirable to perform single-molecule studies directly on proteins present in cell lysates or peripheral blood samples. The proteins obtained in this way more faithfully represent post-translational modifications important for their functionality.

**Fig. 5:**
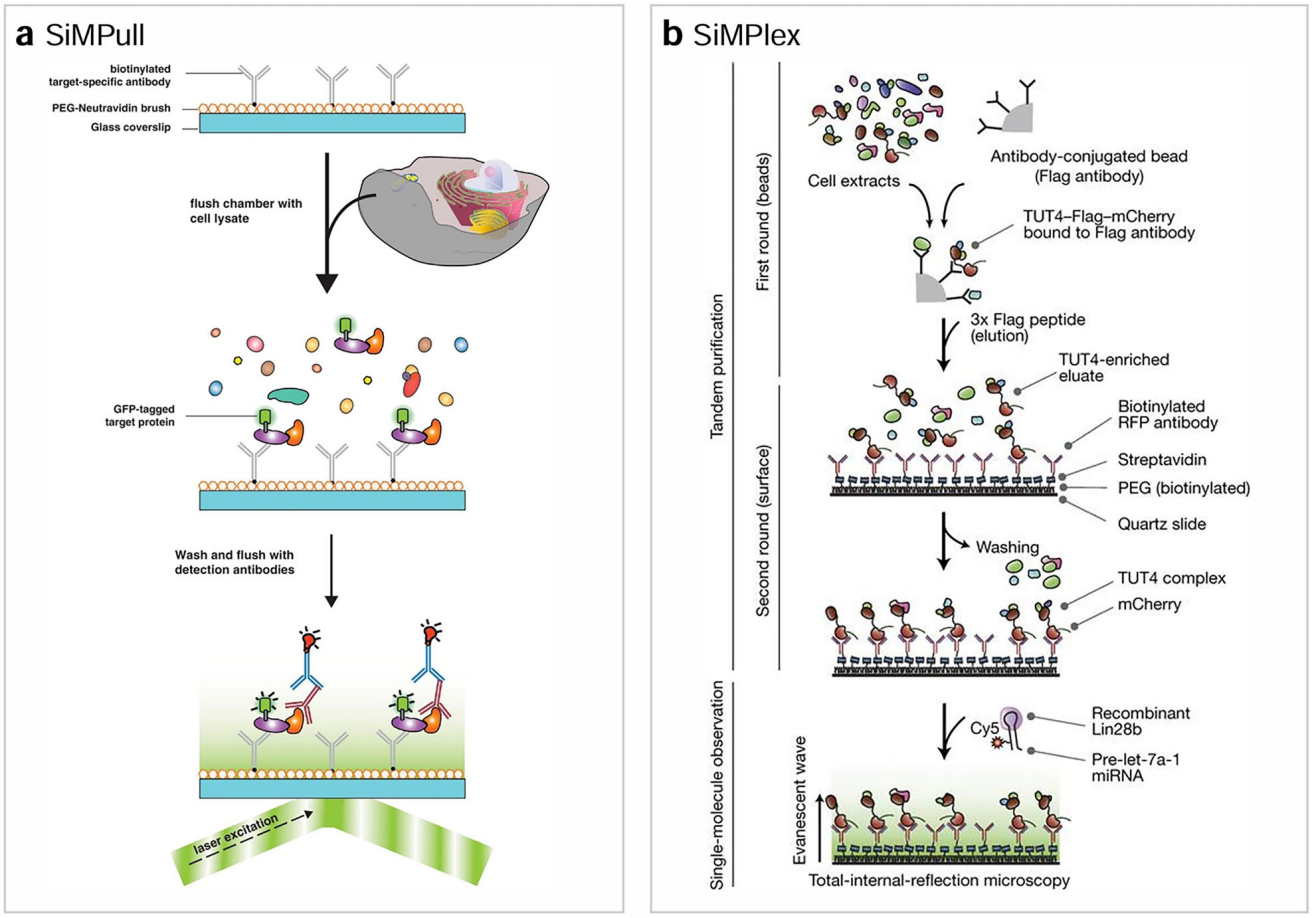
Single-molecule fluorescence techniques for protein detection. **a**, **b** The schematics of SiMPULL^66^ (**a**) and SiMPlex^43^ (**b**).

Techniques such as single-molecule pull-down assay (SiMPull, Fig. 5a), single-molecule approach to immunoprecipitated protein complexes (SIMPlex, Fig. 5b) and single-molecule coimmunoprecipitation (single-molecule co-IP) were developed to address this challenge^42–44^. In these techniques, a protein of interest (bait) is captured on a surface using an antibody or a molecule with a high binding affinity to a tag fused to the bait protein. The bait protein brings along its binding partners (prey) during surface immobilization, or its transient interactions with its binding partners can be studied at later stages. The bait and prey proteins can be detected at the single-complex level using fluorescently labeled detection antibodies or a fluorescent protein tag.

Compared to conventional pull-down or co-IP assays, which take many hours from sample preparation to measurement, these single-molecule protein detection techniques require only about 30 min. In addition to stable interactions, transient protein–protein interactions with binding lifetimes of milliseconds can be studied. These transient interactions cannot be investigated via conventional techniques such as western blot or mass spectrometry, which follow the affinity-based copurification of interacting proteins. Furthermore, these techniques can determine the stoichiometry of copurified protein complexes and directly measure the expression levels of protein complexes by simply counting them.

Single-molecule pull-down and co-IP techniques have been successfully used to characterize signaling complexes of the human epidermal growth factor receptor (HER) family^45^ and to predict the targeted drug efficacy of B cell-lymphoma-2 homology-3 (BH3) mimetics using liquid biopsy samples^46^. However, it remains to be seen whether they can be useful in cancer diagnosis in clinical settings.

### Surface-enhanced Raman for single biomarker detection

Many single-molecule techniques rely on fluorescence signals for biomarker detection because these signals are sufficiently strong to be easily detected at the single-molecule level. Recently, Raman scattering, typically considered too weak to be detected at the single-molecule level, has been demonstrated to detect single biomarkers using surface-enhanced Raman scattering (SERS).

**Fig. 6:**
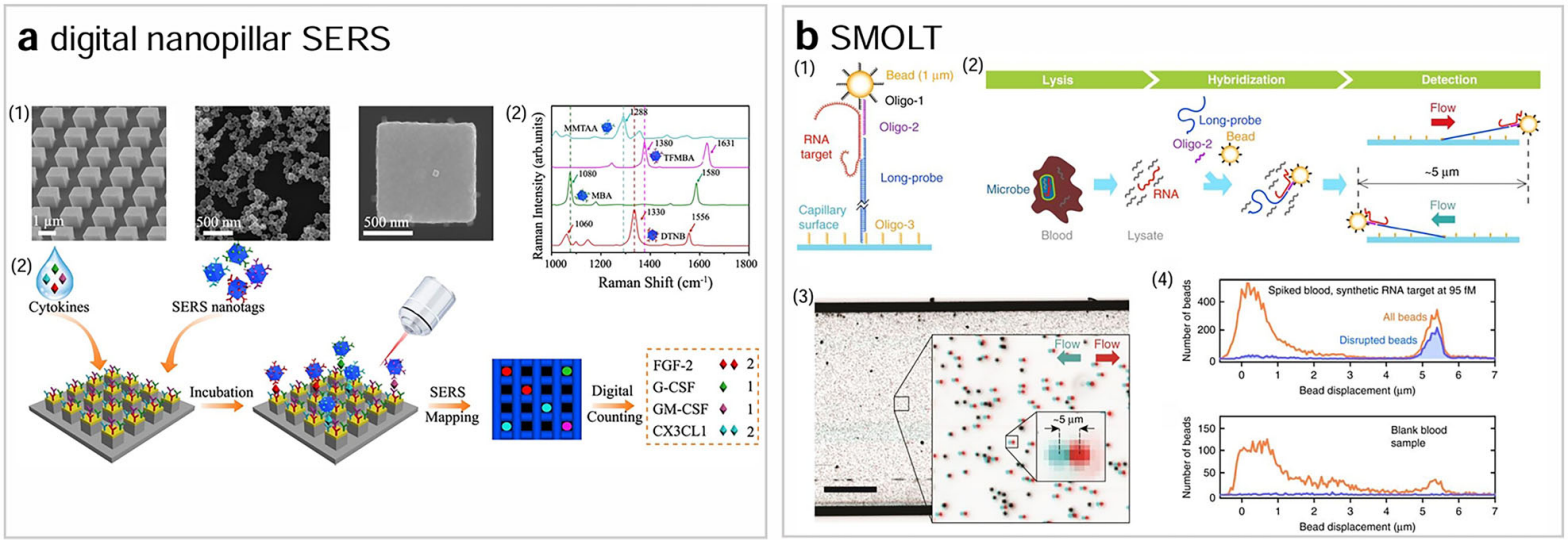
Non-fluorescence single-molecule detection techniques. **a**, **b** The schematics of digital nanopillar SERS^47^ (**a**) and SMOLT^48^ (**b**).

One notable example that utilizes SERS for single biomarker detection is digital nanopillar SERS^47^ (Fig. 6a). In this technique, an array of pillars is fabricated using electron beam lithography, and the tops of the pillars are functionalized with multiple kinds of target-specific antibodies. By controlling the concentration of target molecules, they are captured on the pillars in such a way that most pillars bind to either one or zero target molecule. The target molecules captured on the pillars are then bound by target-specific SERS nanotags, whose surfaces are coated with both Raman reporters and antibodies. The target-specific SERS nanotags and, thus, the target molecules are identified by imaging the nanotags using a Raman microscope. With much narrower spectral peaks of SERS compared to fluorescence (1–2 nm versus 50 nm), digital nanopillar SERS has the potential to excel in multiple protein analysis, though its full realization remains to be seen.

### Magnetic bead-based single biomarker detection

Another method for detecting single molecules involves attaching a micron-size bead to the target molecules and monitoring it using an optical microscope. A notable example of this concept in biomarker detection is a technique called single-molecule tethering (SMOLT)^48^ (Fig. 6b). In SMOLT, magnetic beads are coated with a DNA oligonucleotide that is partially complementary to the target RNA. A long double-stranded DNA probe with a single-stranded overhang, which is partially complementary to a different region of the target RNA, is also used. The magnetic bead and the long DNA probe are connected by hybridizing them with the target RNA. The hybridization complexes are immobilized on the surface of a capillary tube and stretched by a stream of liquid flowing through the tube. The displacement of the beads under flow is determined by the length of the long double-stranded DNA probe specific to the target RNA, allowing different RNAs to be distinguished by their measured displacement.

Without target amplification, SMOLT can detect biomarkers faster than PCR and permits accurate quantification. Another advantage of SMOLT over fluorescence-based techniques is that it requires only simple equipment, such as a low-magnification lens and a low-cost digital camera, for single-molecule detection. However, SMOLT’s LoD is three orders of magnitude worse than that of PCR. Moreover, its multiplexing capacity is limited to a few biomarkers, and it cannot distinguish point mutations with high specificity. These limitations hinder the widespread application of SMOLT in molecular diagnostics.

## Bona fide single-molecule sequencing

The increased speed and throughput of NGS have enabled the rapid detection of multiple biomarkers. The substantial reduction in sequencing costs has made NGS technologies widely accessible for routine medical applications, particularly in precision medicine. However, NGS technologies have several limitations. The library preparation and amplification steps before sequencing are time consuming and prone to bias and errors. Notably, the short-read sequencing lengths of NGS come with inherent limitations, such as inaccurate genome assembly, and the inability to detect large structural variants and variants located in highly repetitive areas. To address these issues, several sequencing technologies have been introduced.

### Helicos sequencing

**Fig. 7:**
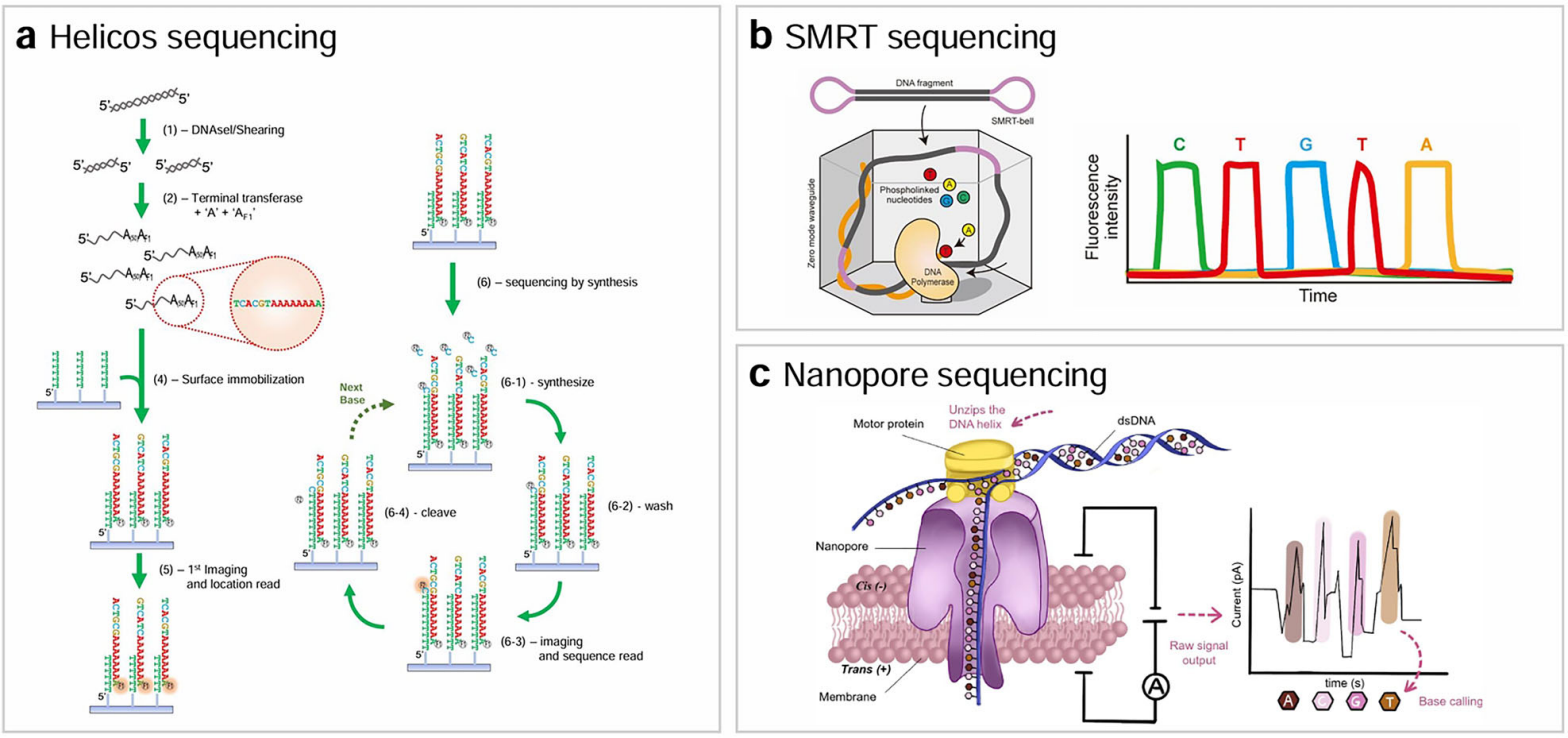
Third-generation sequencing. **a**–**c** The schematics of Helicos sequencing (**a**) SMRT sequencing^67^ (**b**) and Nanopore sequencing^68^ (**c**).

Helicos Biosciences developed a pioneering technology for sequencing individual DNA molecules directly, eliminating the need for amplification^49,50^ (Fig. 7a). This technique, known as true single-molecule sequencing, involves fragmenting DNA into smaller pieces, denaturing it, adding a poly(A) tail to the 3′-end and capturing it on a surface through hybridization with poly(T) DNAs coated on the surface. Fluorescently labeled nucleotides are added one at a time to the detection chamber, and their incorporation into the growing DNA strand is detected using single-molecule fluorescence imaging. After each nucleotide addition, the dye–nucleotide linker is chemically cleaved to release the dye label. The process is then repeated with the addition of a different nucleotide and subsequent imaging, enabling the precise determination of the DNA sequence.

Helicos sequencing was the first true single-molecule sequencing technology, but it could not withstand market competition due to several limitations. It was not ‘real-time’, relying instead on a ‘sequencing by synthesis’ method similar to NGS. The technology offered short-read sequencing with an average read length of around 30 bases. In addition, the sequencing instrument was large and costly, with a price around US$1 million.

### SMRT sequencing

Single-molecule real-time (SMRT) sequencing is a real-time, long-read sequencing technique developed by Pacific Biosciences^51^ (Fig. 7b). In this technique, a library of single-stranded circular DNA templates is created by attaching hairpin adapters to both ends of target double-stranded DNA. Primers bind to the adapters to initiate the polymerase reaction. The library is then loaded into a flow cell containing nanometer-sized wells known as zero-mode waveguides^52^. A complex of single DNA template and polymerase is anchored to the bottom of the waveguide, and fluorescently labeled nucleotides are added. Each of the four nucleotides is labeled with a different fluorophore. The color and duration of the emission are recorded in real time as the polymerase incorporates the fluorescently labeled nucleotides. Because the polymerase is located at the bottom of the zero-mode waveguide, highly enhanced signals can be collected even under high background. After a nucleotide is incorporated, the fluorophore is cleaved and diffuses out. This process occurs simultaneously in many parallel wells in the flow cell.

SMRT sequencing offers an advantage over NGS technologies with its ability to read long sequences (less than 300 bp versus more than 10 kbp). This capability allows SMRT sequencing to provide more reliable data for identifying large structural variants in cancer and detecting repeat expansions associated with genetic diseases such as Huntington’s disease and Fragile X syndrome. Despite these benefits, SMRT sequencing faces several challenges before it can be widely adopted in diagnostics. First, the instruments required for SMRT sequencing are large and costly. Second, it has a higher error rate and lower throughput compared with NGS technologies.

### Nanopore sequencing

Nanopore sequencing is a real-time, long-read sequencing method that does not use fluorescent signals^53–55^ (Fig. 7c). Although the concept of nanopore sequencing has been around since the 1980s^56,57^, it became a practical sequencing method after its commercialization by Oxford Nanopore Technologies in 2014. In this technique, an electrically resistant membrane containing nanometer-sized protein pores is immersed in an electrolytic solution. With a constant voltage applied across the membrane, an ionic current is produced through the nanopore. When single-strand nucleic acids are captured and passed through the nanopores by electrophoresis, the ionic current is limited because the pore is obstructed by nucleobases. Since different nucleobases obstruct the pore to different extents, they can be identified by monitoring the ionic current changes. A crucial aspect of this technique was slowing down the translocation speed of nucleotides, which was achieved by adding a motor protein that moves the nucleic acid molecule through the nanopore in a step-wise manner.

Similar to SMRT sequencing, nanopore sequencing can read sequences longer than 10,000 base pairs using an inexpensive and portable machine. Nanopore sequencing holds great potential for clinical applications in liquid biopsy^58–61^. However, its higher error rate and lower throughput compared to NGS technologies limit its widespread adoption in diagnostics.

## Concluding remarks

Identification or quantification of nucleic acid or protein biomarkers forms the basis of molecular diagnosis for various diseases. The simplest conceptual approach to this goal is to digitally count the number of biomarkers in a given specimen volume, a task not achievable with traditional PCR and ELISA. With the advent of digital PCR and digital ELISA, which amplify target and signaling molecules, respectively, it eventually became possible to count single-molecule biomarkers. Finally, advances in detector sensitivity and novel detection schemes now permit the detection of single-molecule biomarkers without the need for amplification of targets or signals.

Digital PCR and digital ELISA offer several advantages over conventional PCR and ELISA, such as quantitation without the need for a calibration process and enhanced analytic sensitivity. However, the full potential of single-molecule biomarker detection in molecular diagnostics can only be realized with true single-molecule detection techniques. Without an amplification process, faster and more accurate quantitation is possible. The capability of real-time monitoring permits the detection of transient binding events between probes and target biomarkers, eliminating the need for stable biomarker–probe interactions. This capability is crucial for achieving improved analytic specificity and multiplexing. By leveraging the nature of probe–target interactions, nonspecific and specific binding can be more clearly distinguished. As the biomarker–probe interactions are transient, multiple biomarkers immobilized on the same detection chip can be sequentially detected by simply exchanging probes. These advantages, however, come with challenges. Without amplification, the analytic sensitivity of true single-molecule biomarker detection techniques is usually low. One possible solution to this problem is preamplification of target nucleic acids before detection or the active immobilization of target molecules using an electric field, for example.

True single-molecule biomarker detection techniques offer many advantages over conventional diagnostic methods, but several challenges must be addressed to make them practical and routine in medicine. Currently, their capabilities are demonstrated at the laboratory level and need validation through large-scale retrospective and prospective clinical trials. Test results should be repeatable, reproducible, stable and robust. The diagnostic process must be standardized. Finally, instruments for single-molecule detection need to be compact, automated and affordable for widespread adoption in clinical settings. In this context, recent efforts to develop more cost-efficient and higher-throughput single-molecule fluorescence microscopes^62^ are highly commendable and should be further encouraged.
